# Telomere-to-Telomere Genome Assembly of Bursaphelenchus okinawaensis Strain SH1

**DOI:** 10.1128/MRA.01000-20

**Published:** 2020-10-22

**Authors:** Simo Sun, Ryoji Shinya, Mehmet Dayi, Akemi Yoshida, Paul W. Sternberg, Taisei Kikuchi

**Affiliations:** a Department of Infectious Diseases, Faculty of Medicine, University of Miyazaki, Miyazaki, Japan; b School of Agriculture, Meiji University, Kawasaki, Japan; c Forestry Vocational School, Duzce University, Duzce, Turkey; d Laboratory of Genomics, Frontier Science Research Center, University of Miyazaki, Miyazaki, Japan; e Division of Biology and Biological Engineering, Caltech, Pasadena, California, USA; Vanderbilt University

## Abstract

Bursaphelenchus okinawaensis is a self-fertilizing, hermaphroditic, fungus-feeding nematode used as a laboratory model for the genus *Bursaphelenchus*, which includes the important pathogen Bursaphelenchus xylophilus. Here, we report the nearly complete genome sequence of *B. okinawaensis*. The 70-Mbp assembly contained six scaffolds (>11 Mbp each) with telomere repeats on their ends, indicating complete chromosomes.

## Abstract (precis)

Bursaphelenchus okinawaensis is a self-fertilizing, hermaphroditic, fungal-feeding nematode used as a laboratory model for the genus *Bursaphelenchus*, which includes the important pathogen Bursaphelenchus xylophilus. Here, we report the nearly complete genome sequence of *B. okinawaensis*. The 70-Mbp assembly contained six scaffolds (>11 Mbp each) with telomere repeats on their ends, indicating complete chromosomes.

## ANNOUNCEMENT

Bursaphelenchus okinawaensis is a fungus-feeding nematode associated with longhorn beetles (Monochamus maruokai) and the beetles’ host trees (1). Because these nematodes self-fertilize, *B. okinawaensis* has recently emerged as a laboratory model for the genus *Bursaphelenchus* (2), which includes the important plant pathogen Bursaphelenchus xylophilus (3). Here, we generated a nearly complete genome sequence of *B. okinawaensis*.

Botrytis cinerea grown on autoclaved barley grains was fed to *Bursaphelenchus okinawaensis* strain SH1 (maintained at Meiji University) for 13 days. Mixed-stage worms were collected using a modified Baermann funnel technique (4). Briefly, worm culture was suspended in distilled water (dH_2_O) complemented with streptomycin, amphotericin B, and penicillin (antibiotic/antimycotic [anti/anti]; Gibco), and live worms were passed through a sieve lined with Kimwipes (Crecia) followed by discontinuous sucrose gradient centrifugation to remove culture debris (5). Cleaned worms were incubated in a worm lysis solution (buffer G2 with 0.8 mg/ml proteinase K [Qiagen], 50 mM dithiothreitol [Wako], and 0.5 mg/ml RNase A [Invitrogen]; 55°C; 4 h) following two freeze-thaw treatments. High-molecular-weight genomic DNA was extracted via phenol-chloroform extraction and ethanol precipitation. A Nanopore library was prepared from 1 μg of genomic DNA using a ligation sequencing kit (SQK-LSK109; Oxford Nanopore Technologies) according to the manufacturer’s protocol. A single 24-h sequencing run was performed with an R9.4.1 MinION flow cell; thereafter, 2.8 Gbp of sequence data (173,396 reads; *N*_50_, 33.6 kbp) was obtained. The Nanopore reads were base called to generate FASTQ files using the Guppy v4.0.15 basecaller (Oxford Nanopore Technologies) with the supplied dna_r9.4.1_450bps_hac configuration and were quality checked using NanoPlot v1.31.0 (6). An Illumina sequencing library was separately prepared from 1 μg of extracted DNA using a TruSeq DNA sample preparation kit (Illumina) according to the manufacturer’s protocol. The library was sequenced on an Illumina HiSeq 2000 instrument according to the manufacturer’s protocol, producing 101-bp paired-end reads (4.2 Gbp). Raw Illumina sequence data were subjected to the Real-Time Analysis (RTA) v1.12.4.2 analysis pipeline (Illumina). A Hi-C library was prepared from ∼10,000 fresh worms using an Arima-HiC kit (Arima Genomics) followed by a Collibri ES DNA library prep kit (Thermo Fisher Scientific) according to the manufacturers’ protocols and was sequenced using a MiSeq system with the MiSeq reagent kit v3 (101 cycles × 2), and the 3.6 million short reads were quality checked using the Hi-C quality control pipeline (https://phasegenomics.github.io/2019/09/19/hic-alignment-and-qc.html).

Nanopore long reads were assembled using Flye v2.7.1 (7) with the following parameters: --genome size 70 M and -iteration 4. The Flye assembly was highly contiguous, comprising eight long (1- to 12-Mb) contigs and one short (6-kb) contig. Base correction was then performed with the Illumina paired-end reads with two rounds of Pilon v1.23 (8). To scaffold contigs and confirm assembly fidelity, we performed Hi-C analysis on the Flye assembly using the 3D-DNA pipeline v180114 with the following parameters: -g 50 -r 2 --editor-coarse-resolution 2500000 --editor-coarse-region 10000000 --editor-fine-resolution 1000000 --polisher-input-size 20000000 --splitter-input-size 20000000 (9). Juicebox v1.11.08 (10) was used for visualization of the Hi-C results.

**FIG 1:**
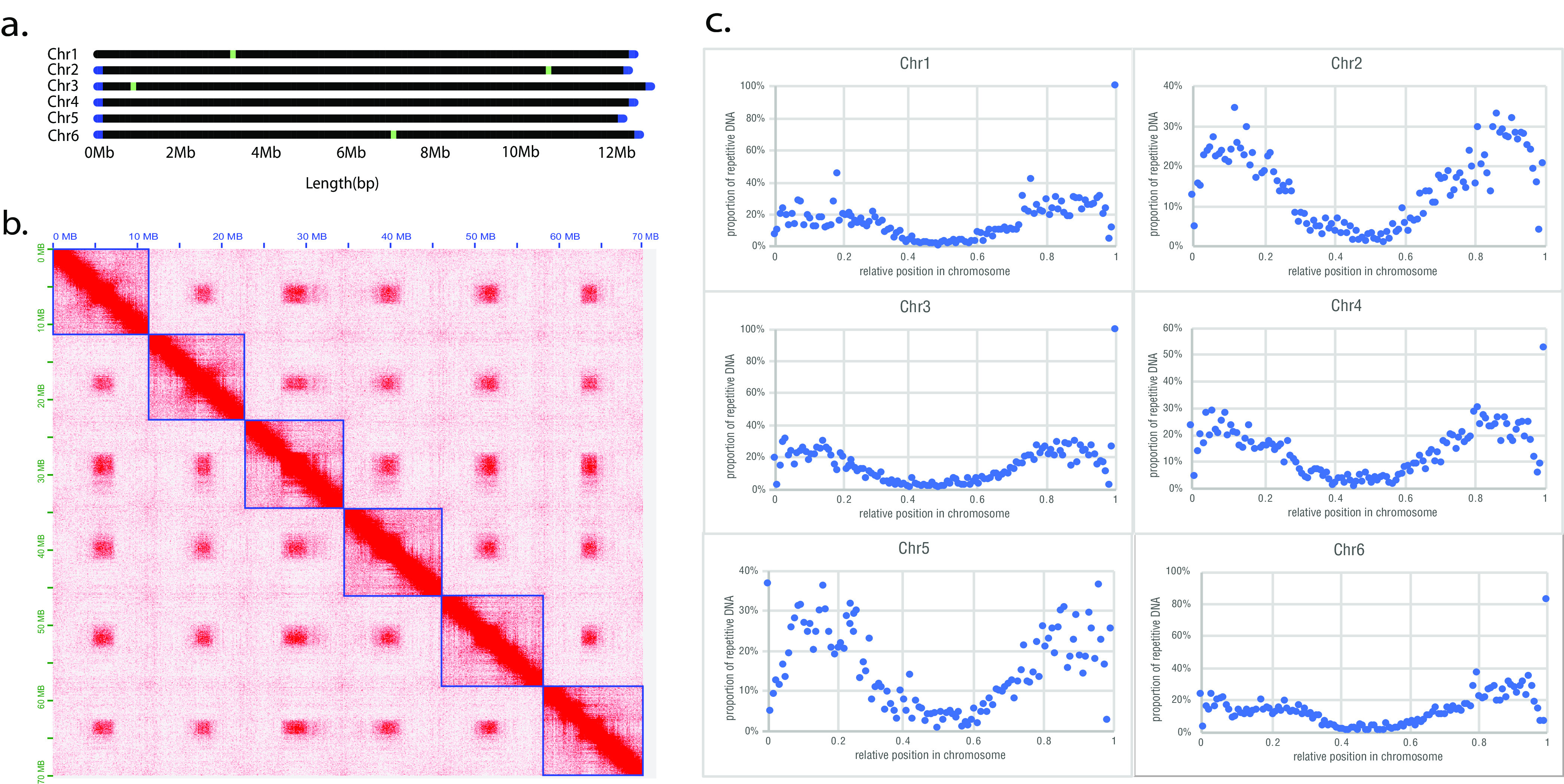
Six chromosomes of the *Bursaphelenchus okinawaensis* genome assembly. (a) Scaffold map of the 70-Mbp *B. okinawaensis* genome. Blue boxes represent telomere repeat signatures, and green boxes indicate remaining gaps in the assembly. (b) Hi-C contact map representing 6 chromosomes (blue boxes) of the *B. okinawaensis* genome. (c) Nonrandom distribution of repetitive DNA sequences in the 6 chromosomes. A species-specific repeat library was generated using RepeatModeler v1.0.8 (https://github.com/Dfam-consortium/RepeatModeler) with the default options, and repeats were identified using RepeatMasker v4.0.9 (http://www.repeatmasker.org/) with the default options.

The resulting 70.0-Mbp assembly had a GC content of 36.2%. It comprised six scaffolds (>11 Mbp each) with four gaps and one ∼6-kbp contig. BUSCO v3.1 completeness analyses (11) of the assembled genome revealed that 92.1% of core eukaryote genes are present in this assembly. Notably, we identified a telomere repeat signature (TTAGGC)n at both ends of five scaffolds, indicating complete chromosomal sequences at one end of the sixth scaffold (Fig. 1).

### Data availability.

The *B. okinawaensis* v2 assembly has been deposited in DDBJ/EMBL/GenBank under BioProject number PRJEB40023. The raw Illumina, Nanopore, and Hi-C read data are available in the Sequence Read Archive with accession numbers DRR243691, DRR243689, and DRR243690, respectively.
